# Exploring the Moderation Effect of Educational Stage on Visual Magnocellular Functioning Linked to Reading: A Study in French Primary School Children

**DOI:** 10.3390/children8020068

**Published:** 2021-01-21

**Authors:** Stéphanie Bellocchi, Virginie Leclercq

**Affiliations:** Université Paul Valéry Montpellier 3, Université Montpellier, EPSYLON EA 4556, F34000 Montpellier, France

**Keywords:** reading development, visual magnocellular system, moderation, educational stage

## Abstract

Many studies have investigated the visual magnocellular system functioning in dyslexia. However, very little is known on the relationship between the visual magnocellular system functioning and reading abilities in typical developing readers. In this study, we aimed at studying this relationship and more specifically the moderation effect of educational stage on this link. We thus tested 82 French typical developing readers (40 beginning readers—Grade 1 and 42 advanced readers—Grade 5) with reading tests and a coherent dot motion task measuring the visual magnocellular functioning. Results indicate positive correlations between visual magnocellular functioning and reading for beginning readers but not for advanced readers. Moreover, moderation analyses confirm that reading proficiency moderates the relationship between magnocellular system functioning and reading outcomes. We concluded that the relationship between visual magnocellular pathway functioning and reading abilities in typical developing readers could depend on reading proficiency.

## 1. Introduction

Reading is a central ability for children to develop. A lot of studies identified fundamental linguistic predictors in learning to read, such as phonological awareness, phonological decoding, verbal short-term memory, lexical access, and orthographic processing (e.g., [1,2,3,4]). However, reading also requires the processing of visual information. Consequently, to successfully decode written words children need to develop good visual skills, specifically, visuo-attentional skills (e.g., [1,5,6,7]).

Particularly, several researches indicated that visual attention plays an important role in reading acquisition. Plaza and Cohen [8] explored the development of different reading predictors (i.e., phonological processing, naming speed, and visual attention) in French kindergarten and their contribution to reading and spelling in Grade 1. The major findings revealed that not only syllable awareness, but also visual attention were the most important predictors of early reading and spelling. Moreover, different researchers highlighted a relationship between visuo-spatial attention abilities and reading level in children [5,9,10,11]. More important for our research, it has been shown that the role of attention in reading evolves with expertise, i.e., less attention is needed to process words in expert readers compared to beginning readers [7,12,13]. One possible explanation is that beginning readers, compared to more proficient ones, have to parse words in small units during the phonological decoding [7]. Consequently, visuo-spatial attention is fundamental in beginning readers: (1) to focus attention on small units in words in order to select some graphemes and filtered out other graphemes allowing the essential letter-to-speech sound integration and (2) to orient attention from the beginning to the end of the letter string (e.g., [7,14,15,16]).

Importantly, several studies indicate that visuo-attentional and visuo-spatial skills linked to reading are partly related to the functioning of the visual magnocellular pathway [17,18]. The role of this pathway in reading has been highly studied with dyslexic children leading to the magnocellular theory of dyslexia proposing that difficulties in learning to read are linked to a deficit in the magnocellular visual pathway (e.g., [19,20,21,22,23,24,25,26,27,28]; see Joo, Donnelly, and Yeatman [29] for an alternative hypothesis; see Stein [18], for a recent review). In this vein, Facoetti [16] proposed that a weakened magnocellular input to the dorsal visual stream that mainly controls spatial attention by the occipito-parieto-frontal system could be a possible neurobiological basis for the deficits in some attentional processes in dyslexic readers. In support of this, in dyslexic children, several visuo-spatial and visuo-attentional difficulties have been observed, including asymmetric attention distribution, difficulty in orienting attention [30], deficit in attentional shifting ([15,16,31,32,33] for reviews), and increased crowding [34,35,36,37]; see Bellocchi, Muneaux, Bastien-Toniazzo, and Ducrot [38] for a review). Therefore, the visual magnocellular pathway, that mediates motion perception and object localization, could play a major role in directing visual attention and in eye movement control (e.g., [16,17,18]). Consequently, in reading it would play an important role to rapidly focus ventral stream attention on letters and also to parse sequences of letters by ordering the shifting of visual attention and the eye movements during reading [15,18]. 

However, although many studies have been conducted on the link between the functioning of the magnocellular pathway and reading impairments, few studies have been run specifically on typically developing children while learning to read. In preschool children, Kevan and Pammer [39] found that measures of the dorsal stream functioning can predict emerging reading abilities (letters and words identification) in Grade 1. Barnard and collaborators [40] did not observe a difference in the magnocellular function between good and poor readers in a group of children aged between 4 and 13-year-old. On the contrary, in 10-year-old normal readers, Talcott et al. [41] found a link between visual sensitivity to coherent movement and the ability to extract orthographic information during reading. They thus proposed that sensitive visual magnocellular processing might be important for orthographic aspects of reading. Piotrowska and Willis [42] showed that global motion sensitivity contributes to a small percentage of variance in reading tasks (text reading fluency; word and pseudoword reading) after accounting for age, phonological awareness, non-verbal intelligence, and socio-economic status in a group of children aged between 6 and 11-year-old. Finally, Kinsey and collaborators [43] studied the relationship between attentional processing mediated by visual magnocellular and reading abilities in a group of children aged between 8 and 11-years-old. They found a relationship between visual magnocellular and reading, but stronger for pseudowords compared to irregular words. This result suggested that attentional processes mediated by visual magnocellular functioning have different contributions according to the type of linguistic item to read. Indeed, according to the dual-route model (see [14] for a review), in alphabetic languages unfamiliar words and pseudowords are processed by the sublexical route, based on grapheme-to-phoneme correspondences. It has been shown that stimuli processed by the sublexical route require serial attentional graphemic parsing (e.g., [7,44]). This could explain the stronger link between visual magnocellular functioning and sublexical route, i.e., pseudoword reading (e.g., [30,45]). Furthermore, some evidences suggested that the dorsal stream is at first engaged in the analytic processing necessary for learning to integrate orthographic with phonological and lexical–semantic features of words [46]. 

Summing up, the results of the studies conducted on normal readers suggested a relationship between visual magnocellular functioning and reading abilities. However, they did not explore the effect of educational stage on the relationship between visual magnocellular functioning and reading abilities. Yet, as mentioned above, attentional processes specific to reading evolve with expertise in reading. 

## 2. The Present Study

Given that knowledge is lacking in the literature, the aim of our work is to explore the relationship between the visual magnocellular functioning and reading in typical developing readers. In particular, we wanted to explore the different impact of the visual magnocellular functioning on reading of Grade 1 readers (6-year-old) and Grade 5 readers (10-year-old). Note that we took into consideration two educational stages corresponding to two contrasting reading levels, i.e., Grade 1 corresponding to beginning readers and Grade 5 corresponding to advanced readers. According to the findings showing an important role of visuo-attentionnal processes in beginning reading, (e.g., [7,8]), we predicted that visual magnocellular functioning will be related to reading skills during beginning stages of reading (Grade 1), compared to advanced stages (Grade 5). We tested this prediction using a moderation analysis. Indeed, moderation analysis allows to test for the influence of a third variable (i.e., moderator—Z), on the relationship between two variables (i.e., X and Y). Particularly, moderation tests for when or under what conditions an effect occurs. In our study, educational stage (Grade 1 and Grade 5) corresponded to the moderator variable, visual magnocellular functioning to X variable, and reading outcomes to Y variable. We thus aimed at testing if the educational stage could moderate the relationship between X (visual magnocellular functioning) and Y (reading skills). To the best of our knowledge, this is the very first study using moderation analyses to explore this research question. 

To test this hypothesis, we tested children in Grades 1 and 5 with a series of tasks: the Coherent Dot Motion (CDM) task measuring visual magnocellular pathway functioning and three reading tasks (text, word, and pseudoword reading).

## 3. Method

### 3.1. Participants

Forty-two 6-year-olds Grade 1 readers and forty-five 10-year-olds Grade 5 readers were recruited from two mainstream primary schools in a city in southern France. All children were native speakers of French and had normal or corrected-to-normal vision. (The participants’ vision was not directly tested by the investigators, but their teachers indicated which children wore glasses for reading and these children were asked to perform the experimental tasks with their glasses. Moreover, Grade 1 children’s vision was tested during the previous school year throughout the annual planned medical visit.) None of them suffered from any neurological, psychiatric, or emotional disorders or were educationally disadvantaged. Additionally, none of them were considered by their teachers as either having learning, cognitive, or behavioral difficulties (i.e., children who exhibited attentional or other behavioral problems in class) and we did not include children having neurodevelopmental disabilities (i.e., specific language impairment). They were all tested six months after the beginning of the school year. On the basis of the performance at the Alouette-R test [47], we calculated a raw score for each participant which represents the number of words read per minute (WPM). First, participants who obtained a score outside the interval [average − 3 SD; average + 3 SD] were rejected, then participants who obtained a score outside the interval [average − 2 SD; average + 2 SD] were rejected. Consequently, we excluded two Grade 1 and three Grade 5 children. Also, on the basis of the performance at the CDM_LL test (see below), first, participants who obtained a score outside the interval [average − 3 SD; average + 3 SD] were rejected, then participants who obtained a score outside the interval [average − 2 SD; average + 2 SD] were rejected. Thus, we excluded three Grade 1 and one Grade 5 children.

Consequently, statistical analyses were conducted on thirty-seven 6-year-olds Grade 1 readers (age in months: M = 80.92; SD = 4.11; 20 girls, 17 boys) and forty-one 10-year-olds Grade 5 readers (age in months: 128.39; SD = 4.27 months; 24 girls, 17 boys). All but four 6-year-olds and five 10-year-olds were right-handed.

The study was conducted according to the guidelines of the Declaration of Helsinki [48], and approved by the Institutional Review Board of the Local Education Authority of the Montpellier Academy (France) (31 January 2019). The children’s parents gave their written consent for participation. 

### 3.2. Materials

#### 3.2.1. Reading Abilities

##### Text Reading

We used the Alouette-R test [47]. This is a French standardized test for children aged from 6- to 16-years-old. Children are instructed to read aloud the text composed by 265 words as quickly and accurately as possible. The text contains real words and grammatically correct sentences, but it does not have any meaning. The test provides two raw scores: reading accuracy and reading fluency.

##### Word and Pseudoword Reading

Word and pseudoword reading abilities were assessed with two different standardized tasks, according to children’ educational stage. However, the different tasks provided the same score, i.e., reading fluency that we used in the analyses. 

Children attending Grade 1 were tested with the test called “Reading aloud of familiar words and invented words—session 2” [49]. This test contains two lists of items: one list of 60 regular words and one list of 60 pseudowords. Each list has to be read as quickly and accurately as possible in a limit of time of 1 min. The number of items read in one minute or the time to read the 60 items was recorded for each list. Thus, the test provides a raw score of reading fluency.

Children attending Grade 5 were tested with the reading test from the “Odedys 2” [50]. In this test, three lists of stimuli are presented to the children. A list of 20 regular words, a list of 20 irregular words, and a list of 20 pseudowords. Children were asked to read the 20 successive items, as quickly and accurately as possible. The number of items correctly read and the reading time of the 20 items were recorded for each list. From these scores, a score of reading fluency (number of words/pseudowords correctly read in one minute) was also calculated.

#### 3.2.2. Visual Magnocellular Functioning

##### Coherent Dot Motion (CDM) Task

In this task, children were asked to discriminate the direction of dots motion. Dots can move in four different directions (left, right, upward or downward, 25% each) with two different levels of coherence, randomly intermixed, either a low level of coherent motion (LL) (10%) or a high level of coherent motion (HL) (40%) [23].

A Dell Latitude 5580 computer running MatLab Version R2015_b (MathWorks, Natick, MA, USA) and Psychtoolbox Version 3 [51,52] was used for stimulus generation and experiment control. Stimuli were presented on a 15 inches CRT monitor with a resolution of 1920 × 1080 and a refresh rate of 100 Hz. Participants sat with their eyes approximately 60 cm from the screen. The backgrounds of all displays were a mid-gray (luminance of 90 cd/m^2^). Each trial began with the presentation of a red dot fixation point. After 500 ms, white dots (luminance of 251 cd/m^2^), each subtending a visual angle of 0.06 degrees appeared on the grey background. Dots were contained in a circle of 12° of diameter and their number was approximately 10 per deg^-2^ at each frame (duration = 16.7 ms). The dots density remained constant throughout the trial using the Shadlen–Movshon algorithm with limited lifetime of three frames [53]. Dots speed was 12 °/s. The CDM duration was 400 ms. After the presentation of the dots, a response screen with four lines (one for each possible direction of the dots) was presented. Participants were asked to discriminate the direction of dots movement (upward, downward, leftward, or rightward) by clicking with the mouse on one of the four lines. The response screen was presented until participant answered but with a maximum time of 4 s. Only response accuracy was collected (we specified to the participants that response speed was not relevant). The experimental session consisted of 80 trials (40 trials for each coherence level with 10 for each direction per coherence level) and the succession of trials was randomized. The test was carried out following 8 practice trials to assure that children fully understand the expected task. Compared to the test trials, feedback was proposed on practice trials. If the experimenter had any doubts about the children’s comprehension of the task, another round of eight practice trials was presented. Breaks were proposed every 10 trials. For the CDM low level (CDM_LL) and high level (CDM_HL) of coherence percentage of correct response was calculated.

### 3.3. Data Selection and Analyses 

In order to explore the relationship between the scores obtained at the CDM task and those obtained at the reading tasks, we run bivariate correlations (Pearson) on raw scores. This analysis was separately run for each educational stage. 

Afterwards, to investigate the moderation effect of educational stage on this relationship, we performed moderation analyses using the PROCESS macro developed by Hayes [54] for the SPSS^®^ program, version 21.0 (IBM, Armonk, NY, USA). With this macro, model 1 was used and we ran 5000 bootstrap resamples to estimate the moderator effects and used the percentile bootstrap method to adjust the confidence interval endpoints. In total, four moderation analyses were conducted, all with educational stage (Grade 1 and Grade 5) as categorical moderator, reading outcomes as Y variable, and CDM performance as X variable. We ran one analysis for each reading score. Moderation analyses were conducted on group’s mean centered X and Y variables with moderator term (educational stage) as a contrast coding variable (−0.5/0.5) [55]. Statistical analyses were conducted using the SPSS^®^ program, version 21.0.

## 4. Results

For each group, Table 1 summarizes means and standard deviations of the raw scores obtained in all the tasks used in the study and means and standard deviations of the z-scores obtained in the standardized reading tasks. For the CDM_LL task, the performances were low, but the performances of Grade 1 and Grade 5 children were significantly different to the chance level (respectively: t (36) = 4.792, *p <* 0.001 and t (40) = 10.602, *p <* 0.001). Also, student tests (one-tailed) were conducted to compare the raw scores of the two groups on reading and CDM tasks (Table 1). As expected, Grade 5 children outperformed Grade 1 children on reading tasks. Also, accordingly to previous results observed in the literature, Grade 1 children obtained significant lower scores compared to Grade 5 children on the CDM task (e.g., [4,56]).

Table 2 and Table 3 present the correlation ratings between all measures, respectively for Grade 1 and Grade 5.

Table 2 shows that in Grade 1, all the measures of the reading tasks correlate with each other. More important for our study, significant correlations emerged between the CDM task and the reading tasks. More precisely, positive correlations were found between CDM_LL condition task and: text reading accuracy (*r* = 0.523, *p* < 0.001), text reading fluency (*r* = 0.549, *p* < 0.001), regular word reading fluency (*r* = 0.374, *p* = 0.022) and pseudowords reading fluency (*r* = 0.397, *p* = 0.015). Significant or trends of significant correlations were obtained between the CDM_HL condition task and the reading tasks. More precisely positive correlation was found between CDM_HL condition task and: regular word reading fluency (*r* = 0.339, *p* = 0.040) and text reading accuracy (*r* = 0.315, *p* = 0.058). However, no significant correlation was found between CDM_HL condition task and pseudoword reading fluency (*r* = 0.283, *p* = 0.089).

As we can see in Table 3, in Grade 5, almost all the measures of the reading outcomes correlate with each other. However, more important for our study, no significant correlations were found between the CDM_LL condition task and the reading outcomes (all *p* = ns). Only one significant correlation was found between CDM_HL condition task and text reading accuracy (*r* = 0.359, *p =* 0.021), but all others correlations were not significant (all *p* = ns).

To summarize, correlation analyses conducted on each grade level seemed to indicate that the relationship between the CDM task and the reading outcomes differed according to children’s educational stage. Indeed, especially with the CDM_LL condition task, correlations with reading scores were found for beginning readers (Grade 1) but not for advanced readers (Grade 5). 

To test the hypothesis that educational stage moderates the effect of performance of CDM task on the reading tasks, four moderation analyses were conducted on group’s mean centered scores. Note that we used group’s mean centered scores in order to avoid a direct impact of age on reading tasks and CDM task. That is, the scores at the CDM task and the scores at the reading tasks were all mean-centered in Grade 1, and all mean-centered in Grade 5. Those mean-centered scores were collapsed together for the moderation analyses. Moreover, as we can see in Table 1, performance in the HL condition of the CDM task was very high, especially in Grade 5. To avoid ceiling effects, we thus performed subsequent analyses only on data obtained in the LL condition. 

Table 4 shows the four linear model for each analysis.

Results indicated that educational stage moderated correlation between the CDM task and text reading fluency, and between the CDM task and pseudowords reading fluency (Table 4).

**Figure 1:**
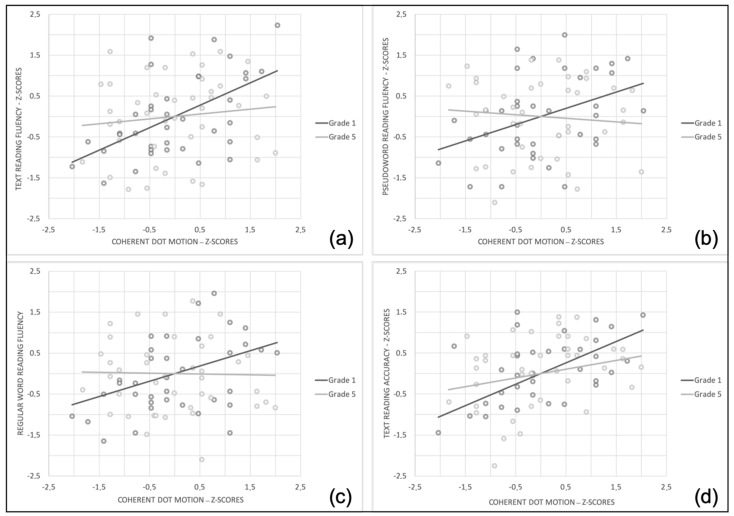
Simple slopes equations of the regression of Coherent Dot Motion z-scores on (**a**) text reading fluency z-scores, (**b**) pseudoword reading fluency z-scores, (**c**) regular word reading fluency, (**d**) text reading accuracy at two levels of educational stages (Grade 1 and Grade 5).

More precisely, for the analysis on text reading fluency, simple slopes analysis (Figure 1a) showed that for Grade 1 as CDM scores increased so text reading fluency score increased, b = 0.548, 95% confidence interval [t = 3.525, *p* = 0.001, (CI; [0.238, 0.859])]. However, for Grade 5, as CDM scores increased, text reading fluency score did not necessarily increase, b = 0.121, t = 0.818, *p* = 0.416, 95% CI [−0.173, 0.415].

With regard to pseudoword reading, simple slopes analysis (Figure 1b) showed that, for Grade 1, as CDM scores increased so pseudoword reading scores increased, b = 0.387, 95% of confidence interval [t = 2.447, *p =* 0.017, (CI; [0.074, 0.720])]. However, for Grade 5, as CDM scores increased, pseudoword reading scores did not necessarily increase, b = −0.087, t = −0.566, *p =* 0.573, 95% CI [−0.394, 0.219].

Finally, results did not indicate that educational stage moderated significantly correlation between the CDM task and text reading accuracy, and between the CDM task and irregular word reading fluency (Table 4 and Figure 1c,d).

## 5. Discussion

Many studies have investigated the visual magnocellular functioning in dyslexia, but few researches have explored the visual magnocellular functioning during normal reading (but see [39,41]). Hence, very little is known on the link between the visual magnocellular functioning and reading development. Our goal was thus to explore whether the link between visual magnocellular functioning and reading outcomes might be affected by educational stage. We hypothesized a higher impact of visual magnocellular functioning on beginning readers’ reading abilities (Grade 1) compared to advanced readers’ ones (Grade 5). We examined this prediction using a moderation analysis. We thus aimed at testing if the educational stage could moderate the relationship between visual magnocellular functioning and reading skills. To the best of our knowledge, this is the very first study using moderation analyses to explore this research question. 

Our results indicated that in Grade 1 the higher the performance to the CDM task, the better the performance of children in reading. Here, the observed relationship between visual magnocellular functioning and reading abilities is in line with the results of Talcott et al. [41], Kinsey et al. [43], and with very recent findings showing correlations between flicker fusion frequency (a measure of M function) and reading performance (across a variety of measures) in typically developing children (aged 8–12) [27]. However, according to our hypothesis, this relationship was found only in Grade 1 (i.e., beginning readers) but not in Grade 5 (i.e., advanced readers). In addition, moderation analyses indicated that the relationships between CDM performances and text and pseudoword reading are significantly moderated by the educational stage. Consequently, our study allows at specifying that the relationship between visual magnocellular functioning and reading changes with reading proficiency. How can we explain this result? In first-grade children, who are starting to learn to read, written language processing is highly demanding in cognitive resources: children have to decode words, that is, to parse lexical unit in sublexical units. Thereby, according to the dual-route model (see [14] for a review), beginning readers mainly process words throughout the sublexical route, based on grapheme-to-phoneme correspondences. This was supported by the results of the correlation analyses showing very strong correlations between regular word reading and pseudoword reading, regular word reading and text reading, and text reading and pseudoword reading, in Grade 1 children. These results might provide evidence that beginning readers were using the same sub-lexical processing across these tasks. With regard to the involved visuo-attentional processes, beginning readers have to process words with a reduced attentional window oriented toward the beginning of the letter string then scanning along the letter string (e.g., [7,57]). Indeed, as stated in the Introduction section, the sub-lexical route is crucial for decoding new words during the first steps of reading acquisition, and it specifically requires serial attentional graphemic parsing (e.g., [7,44]). More importantly, this serial attentional graphemic parsing has been related to visual magnocellular functioning [15,58] for the link between magnocellular pathway and serial search task requiring an attentional spotlight scanning). Consequently, our results suggested that when children are starting to learn to read, visual magnocellular functioning involved in visuo-attentional processes are particularly needed. In other words, it is suggested here that efficient visual magnocellular functioning is important in order to develop good decoding skills in the early stages of learning to read.

To support this interpretation, the pattern of results obtained on fifth-graders is crucial. Indeed, statistical analyses revealed very slight relationship between visual magnocellular pathway functioning and any reading outcomes in Grade 5. With years of reading practice, children reach automatic recognition of words leading to a process of those stimuli by a large attentional window without the need to split lexical units in sublexical units. Accordingly, we found weaker correlations between pseudoword reading and text and word reading compared to beginning readers. This could suggest that Grade 5 children were using different reading-related processes across the text, word, and pseudoword tasks. Consequently, in order to correctly read, it is possible that proficient readers need less visuo-attentional abilities mediated by visual magnocellular pathway to process words compared to beginning readers [7,12,13]. However, in line with this proposal, one could expect a relationship between CDM performances and pseudoword reading in proficient readers since pseudoword reading may require sublexical processing. However, no such significant correlation was found here. One possible explanation is that the pseudowords used in our study were short (there were only three pseudowords with more than two syllables and only seven composed by more than six letters). To decode those short pseudowords, Grade 5 children probably did not engage sublexical processing. Accordingly, in a group of Grade 4 and Grade 5 children, Martens and De Jong [59] did not find a length effect for pseudowords of four to six letters. Length effect is often proposed to reflect a slow and sequential process of reading. The authors proposed that using short pseudowords could prevent such effect from being observed, especially in 4- to 5-Grade normal readers. Nevertheless, a new experiment with longer pseudowords would be appropriate in order to be sure to activate sublexical processing in advanced readers. Since longer pseudowords require attentional processing to spread the sequence of letters into small units, we would expect a relationship between long pseudoword reading and visual magnocellular functioning, even for children in Grade 5.

Our results lead to conclude that the relationship between visual magnocellular functioning and reading changes with reading proficiency. One possible objection to this interpretation is that age, instead of reading proficiency, could explain the differences on visual magnocellular functioning between Grade 1 and Grade 5 children. Indeed, some developmental studies showed that the sensitivity to motion evolves from 6- to 14-year-old [4], while others found that magnocellular sensitivity increased with age and plateaued at around 10 years of age [40,56]. However, our additional analyses (see Appendix A) conducted on a group of poor readers (composed by fourteen Grade 1 and thirteen Grade 5 children) and a group of good readers (composed by ten Grade 1 and thirteen Grade 5 children) indicated that good readers outperformed poor readers on visual magnocellular functioning, regardless participants ‘age. Furthermore, these additional analyses indicated stronger correlations between the CDM scores and reading scores on poor readers compared to good ones, suggesting an higher impact of visual magnocellular functioning on poor readers’ reading abilities compared to good readers’ ones. Even if these complementary results are based on a small number of participants and further experiments to replicate them are required, they clearly suggested that, beyond age, reading proficiency could explain the differences obtained on visual magnocellular functioning.

Finally, we based the development of the CDM task of the present study on the methodology used by Gori et al. [23]. Another way to measure motion sensitivity with this task is to measure the threshold at which children perceive the motion of the points above the chance level. This method could allow a more accurate measurement of motion sensitivity since it is adapted to each child. It would be thus interesting to replicate the results of the present study by using this alternative method. However, since the sensitivity threshold method is time-consuming compared to the one we used, it would be important, at first, to find a solution to make it more feasible with young children who have to be assessed in many other abilities. Also, several studies showed that beyond phonological skills, visuo-attentional abilities are predictors of reading development (e.g., [1,8]). In the present study we did not assess additional phonological abilities in order to examine, above and beyond other-well known predictors, such as phonological awareness and rapid naming abilities, whether visual magnocellular functioning accounted for variance in typical reading ability. Additionally, no measure of non-verbal IQ was included in the protocol in order to control for language-free intelligence. These limitations deserve more consideration in future studies.

Summing up, our study showed that the impact of visual magnocellular functioning in reading abilities changes with reading proficiency. Here, our results highlighted a link between visual magnocellular functioning and reading especially in beginning readers.

## Figures and Tables

**Table 1:** Means (standard deviations) of all variables included in the study (raw and z-scores) and assessed in Grade 1 and Grade 5. [CDM_LL = low level (10%) of coherent motion in CDM task; CDM_HL = high level (40%) of coherent motion in CDM task] [CDM= Coherent Dot Motion].

	Grade 1	Grade 5		
	Mean (SD)	Mean (SD)	t (76)	*p*
Regular word reading (accuracy; % of correct responses)				
raw scores	_	91.585 *(5.527)*	_	_
z-scores		−0.157 *(0.614)*		
Regular word reading (speed; s)				
raw scores	_	23.780 *(7.185)*	_	_
z-scores		0.152 *(0.844)*		
Regular word reading (fluency)				
raw scores	39.384 *(14.810)*	50.093 *(14.571)*	−3.216	*<0.001*
z-scores	−0.012 *(0.827)*	−0.003 *(0.729)*		
Irregular word reading (accuracy; % of correct responses)				
raw scores	_	78.780 *(14.040)*	_	_
z-scores		0.159 *(0.802)*		
Irregular word reading (speed; s)				
raw scores	_	26.805 *(11.724)*	_	_
z-scores relative to the norm		−0.039 *(1.292)*		
Irregular word reading (fluency)				
raw scores	_	52.875 *(14.653)*	_	_
z-scores		0.060 *(1.047)*		
Pseudoword reading (accuracy; % of correct responses)				
raw scores	_	82.320 *(11.020)*	_	_
z-scores		−0.190 *(0.958)*		
Pseudoword reading (speed; s)				
raw scores	_	29.439 *(10.092)*	_	_
z-scores		0.320 *(1.023)*		
Pseudoword reading (fluency)				
raw scores	28.784 *(8.616)*	37.264 *(12.110)*	−3.528	*<0.001*
z-scores	0.057 *(0.724)*	0.065 *(0.991)*		
Text reading (accuracy; %)				
raw scores	81.253 *(8.982)*	94.388 *(2.419)*	−9.014	*<0.001*
z-scores	−0.416 (0.998)	−0.153 (0.605)		
Text reading (fluency)				
raw scores	83.108 *(34.374)*	274.206 *(63.462)*	−16.281	*<0.001*
z-scores	0.415 *(1.001)*	0.076 *(0.774)*		
CDM_LL (% of correct responses)				
raw scores	31.284*(7.985)*	47.659 *(13.710)*	−6.355	*<0.001*
CDM_HL (% of correct responses)				
raw scores	59.193 *(22.646)*	89.732 *(11.562)*	−7.609	*<0.001*

**Table 2:** Bivariate correlations between all measures (reading tasks and CDM task) assessed in Grade 1. [CDM_LL = low level (10%) of coherent motion in CDM task; CDM_HL = high level (40%) of coherent motion in CDM task].

	CDM_HL	Regular Word Reading (Fluency)	Pseudoword Reading (Fluency)	Text Reading (Accuracy)	Text Reading (Fluency)
CDM_LL	0.614 ***	0.374 *	0.397 *	0.523 **	0.549 ***
CDM_HL		0.302 °	0.289	0.315 °	0.339 *
Regular word reading (fluency)			0.875 ***	0.626 ***	0.871 ***
Pseudoword reading (fluency)				0.655 **	0.837 ***
Text reading (accuracy)					0.701 ***

*** = Correlation is significant at the 0.001 level (2-tailed). ** = Correlation is significant at the 0.01 level (2-tailed). * = Correlation is significant at the 0.05 level (2-tailed). ° = *p*-value is in the interval [0.05, 0.07] (2-tailed).

**Table 3:** Bivariate correlations between all measures (reading tasks and CDM task) assessed in Grade 5. [CDM_LL = low level (10%) of coherent motion in CDM task; CDM_HL = high level (40%) of coherent motion in CDM task].

	CDM_HL	Regular Word Reading (Speed)	Regular Word Reading (Accuracy)	Regular Word Reading (Fluency)	Irregular Word Reading (Speed)	Irregular Word Reading (Accuracy)	Regular Word Reading (Fluency)	Pseudo-Word Reading (Speed)	Pseudo-Word Reading (Accuracy)	Pseudo-Word Reading (Fluency)	Text Reading (Accuracy)	Text Reading (Fluency)
CDM_LL	0.538 ***	0.037	−0.086	−0.021	0.228	0.035	−0.103	0.075	0.120	−0.087	0.215	0.121
CDM_HL		0.116	0.134	−0.063	0.192	0.071	−0.048	0.170	−0.078	−0.150	0.359 *	0.012
Regular word reading (speed)			−0.164	−0.903 ***	0.857 ***	−0.222	−0.680 ***	0.613 ***	−0.281	−0.712 ***	−0.461 **	−0.782 ***
Regular word reading (accuracy)				0.343 *	−0.284 °	0.332 *	0.289 °	0.193	0.205	−0.073	0.378 *	0.033
Regular word reading (fluency)					−0.723 ***	0.212	0.706 ***	−0.549 ***	0.213	0.669 ***	0.354 *	0.759 ***
Irregular word reading (speed)						−0.377 *	−0.788 ***	0.389 *	−0.294 °	−0.488 **	−0.509 ***	−0.603 ***
Irregular word reading (accuracy)							0.675 ***	0.206	0.463 **	−0.008	0.456 **	0.269
Irregular word reading (fluency)								−0.164	0.280	0.315 *	0.439 **	0.612 ***
Pseudoword reading (speed)									−0.192	−0.916 ***	−0.195	−0.607 ***
Pseudoword reading (accuracy)										0.430 **	0.560 ***	0.257
Pseudoword reading (fluency)											0.328 *	0.696 ***
Text reading (accuracy)												0.458 **

*** = Correlation is significant at the 0.001 level (2-tailed). ** = Correlation is significant at the 0.01 level (2-tailed). * = Correlation is significant at the 0.05 level (2-tailed). ° = *p*-value is in the interval [0.05, 0.07] (2-tailed).

**Table 4:** Linear model for each moderation analysis with 95% CIs.

Variables		b	SE B	t	*p*	95% CI
Regular word reading (fluency) *R*^2^ = 0.067	Constant	0.000	0.111	0.000	1.000	[−0.221, 0.221]
	CDM	0.177	0.103	1.572	0.120	[−0.047, 0.401]
	Educational stage	0.000	0.222	0.000	1.000	[−0.442, 0.442]
	CDM x Educational stage	−0.395	0.225	−1.756	0.083	[−0.843, 0.053]
Pseudoword reading (fluency) *R*^2^ = 0.079	Constant	0.000	0.110	0.000	1.000	[−0.220, 0.220]
	CDM	0.155	0.110	1.386	0.170	[−0.068, 0.378]
	Educational stage	0.000	0.221	0.000	1.000	[−0.440, 0.440]
	CDM x Educational stage	−0.484	0.224	−2.17	0.034*	[−0.929, −0.039]
Text reading (accuracy) *R*^2^ = 0.154	Constant	0.000	0.106	0.000	1.000	[−0.211, 0.211]
	CDM	0.369	0.107	3.445	0.001 *	[0.156, 0.582]
	Educational stage	0.000	0.211	0.000	1.000	[−0.421, 0.421]
	CDM x Educational stage	−0.301	0.214	−1.437	0.155	[−0.735, 0.119]
Text reading (fluency) *R*^2^ = 0.150	Constant	0.000	0.106	0.000	1.000	[−0.211, 0.211]
	CDM	0.335	0.107	3.120	0.003 *	[0.121, 0.548]
	Educational stage	0.000	0.212	0.000	1.000	[−0.422, 0.422]
	CDM x Educational stage	−0.428	0.215	−1.994	0.049 *	[−0.855, −0.000]

* = significant *p*-value.

## Data Availability

Data available on request due to restrictions.

## Appendix A

In order to better understand the impact of reading proficiency, we ran additional analyses. For each group, we calculated the z-scores of the words read per minute score (WPM) of the Alouette-R test [47]. We then ranked all the children according to their reading level and created two groups: a group of “poor readers” (z-score below −0.5 composed of twenty-seven participants: fourteen Grade 1 and thirteen Grade 5 children) and a group of “good readers” (z-score above 0.5 composed of twenty-three participants: ten Grade 1 and thirteen Grade 5 children). Afterward, we ran student tests (one-tailed) in order to compare the two groups at the CDM task, then we ran two separated correlation analyses (one for the group of poor readers and one for the group of good readers) to test the relationship between CDM and reading abilities. Firstly, comparison analyses indicated significant higher scores on the CDM_LL and the CDM_HL for the “good readers” group (respectively, M = 0.436; SD = 0.117 and M = 0.832; SD = 0.252) compared to the “poor readers” group (respectively, M = 0.368; SD = 0.155 and M = 0.708; SD = 0.207) (respectively t(48) = −1,713; *p =* 0.047 and (t(48) = −1.867; *p* = 0.034). Secondly, correlation analyses conducted on the “poor readers” group (Table A1) indicated a correlation between the CDM scores (LL coherence and the HL coherence) and reading scores (except for pseudowords). On the contrary, for “good readers” (Table A2), analyses showed a significant correlation only between the CDM_LL score and the text reading fluency score. No other correlation between CDM scores and reading tasks scores was significant.

**Table A1:** Bivariate correlations between all measures (reading tasks and CDM task) assessed in poor readers [CDM_LL = low level (10%) of coherent motion in CDM task; CDM_HL = high level (40%) of coherent motion in CDM task].

	CDM_HL	Regular Word Reading (Fluency)	Pseudoword Reading (Fluency)	Text Reading (Accuracy)	Text Reading (Fluency)
CDM_LL	0.673 ***	0.368 °	0.259	0.598 ***	0.657 ***
CDM_HL		0.504 **	0.302	0.658 ***	0.679 ***
Regular wod reading (fluency)			0.731 ***	0.702 ***	0.757 ***
Pseudoword reading (fluency)				0.572 **	0.616 ***
Text reading (accuracy)					0.801 ***

*** = Correlation is significant at the 0.001 level (2-tailed). ** = Correlation is significant at the 0.01 level (2-tailed).* = Correlation is significant at the 0.05 level (2-tailed). ° = *p*-value is in the interval [0.05, 0.06] (2-tailed).

**Table A2:** Bivariate correlations between all measures (reading tasks and CDM task) assessed in good readers [CDM_LL = low level (10%) of coherent motion in CDM task; CDM_HL = high level (40%) of coherent motion in CDM task].

	CDM_HL	Regular Word Reading (Fluency)	Pseudoword Reading (Fluency)	Text Reading (Accuracy)	Text Reading (Fluency)
CDM_LL	0.628 ***	0.090	0.049	0.266	0.440 *
CDM_HL		0.011	−0.089	0.311	0.406 °
Regular word reading (fluency)			0.549 **	0.053	0.193
Pseudoword reading (fluency)				0.323	0.598 **
Text reading (accuracy)					0.704 ***

*** = Correlation is significant at the 0.001 level (2-tailed). ** = Correlation is significant at the 0.01 level (2-tailed).* = Correlation is significant at the 0.05 level (2-tailed). ° = *p*-value is in the interval [0.05, 0.06] (2-tailed).
